# Real-life data of adjuvant IFN-α2b and MMC in conjunctival melanocytic lesions

**DOI:** 10.1007/s00417-022-05832-1

**Published:** 2022-10-18

**Authors:** Simone Nuessle, Claudia Auw-Haedrich, Jana Jiang, Daniel Boehringer, Thomas Reinhard

**Affiliations:** Eye Center, Medical Center, Faculty of Medicine, University of Freiburg, Killianstr. 5, 79106 Freiburg, Germany

**Keywords:** Interferon alpha 2b, Mitomycin C, Conjunctival melanoma, Primary acquired melanosis with atypia, Adjuvant

## Abstract

**Purpose:**

We herein compare topical interferon alpha 2b (IFN-α2b) to topical mitomycin C (MMC) in the adjuvant management after excision of primary acquired melanosis with atypia (PAM) and melanoma of the conjunctiva/cornea (CM).

**Methods:**

We included 25 tumors from 25 patients (six with PAM and 19 with CM). After surgical excision, four patients started with adjuvant IFN-α2b (two in combination with radiotherapy), 19 with MMC, and two with radiotherapy alone. Five patients were switched from initial MMC/radiotherapy to IFN-α2b during follow-up. Efficacy was assessed via time to tumor recurrence and initial therapy response.

**Results:**

With initial IFN-α2b, three patients (3/4, two with additional radiotherapy) showed complete remission (follow-up: 1478–1750 days) and one recurrence (1/4) was noted after 492 days. With initial MMC, no recurrence was recorded in 15 of the 19 patients (follow-up: 99–4732 days). Five patients were switched from MMC or radiotherapy to IFN-α2b: two patients showed complete remission (2/5), while another two (2/5) experienced recurrences and remained without recurrence after repeated courses of IFN-α2b (follow-up: 1798 and 1973 days). Only one patient showed incomplete response. Adverse effects were recorded in five patients, all received MMC.

**Conclusion:**

Topical IFN-α2b (arguably together with radiotherapy) may be a viable alternative to MMC in PAM and CM. We observed fewer side effects at similar response rates. However, when response to MMC was poor, IFN-α2b may also be of limited utility.


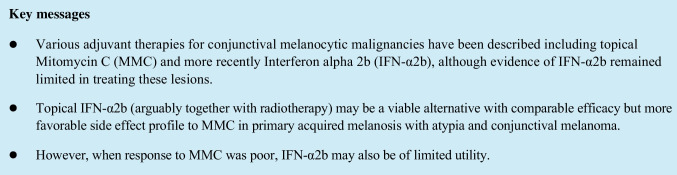


## Introduction

Conjunctival melanoma (CM) is a rare tumor accounting for 0.24% of all melanomas with an incidence of 0.6–0.8 cases/million/year in northern Europe but is one of the most important conjunctival malignancies due to its potentially fatal outcome [1]. Local recurrence was reported to occur from 11 to 69% with a median time of 11–17 months [2].

CM most commonly arises from primary acquired melanosis with atypia (PAM) in up to 75%, but also de novo and from pre-existing nevi. CM and PAM both arise from melanocytes of the conjunctiva [3]. Sometimes, these lesions initially occur on the cornea despite conjunctival origin [4]. PAM is characterized by abnormal intraepithelial melanocytes. A histopathological classification of these melanocytes in having atypia or no atypia is important as the risk for melanoma development in PAM with atypia is almost 50% and PAM without atypia almost never transform [5]. The terminology “PAM with/without atypia” is currently being re-evaluated with respect to prognosis, and the different classification systems (PAM, c-MIN (conjunctival melanocytic intraepithelial neoplasia), and the World Health Organization (WHO) 4th edition classification (c-MIL = conjunctival melanocytic intraepithelial lesion)) are being discussed [6].

The most common treatment is wide local excision using a “no-touch technique”, sclerectomy, and cryotherapy on the margins [7]. However, recurrences especially following incomplete excision are common. Over the years, less-invasive procedures have been increasingly preferred to reduce ocular morbidity. This shift was possible by the use of adjuvant therapies. Besides cryotherapy, these include radiotherapy and topical chemotherapeutic agents such as mitomycin C (MMC) and interferon alpha 2b (IFN-α2b). Topical MMC has been described since 1993 as an effective adjuvant for conjunctival melanocytic malignancies [8, 9]. MMC, a quinone antibiotic with anti-tumor properties, induces cellular apoptosis via DNA damage by alkylation or free radical injury. However, MMC eye drops not only damage tumor cells but also healthy tissue [10]. Therefore, MMC bears the risk of potential long-term ocular surface toxicities with limbal stem cell deficiency [11, 12]. There is no uniform recommendation for application, but most studies favor a treatment in cycles due to the toxic effects following longer therapy duration. In contrast, IFN-α2b eye drops are considered a drug with scant side effects and an effective agent in treating ocular squamous surface neoplasia [13]. It is a naturally occurring cytokine released by immune cells and is thought to suppress tumor growth by enhancing the immune response [14]. Its use in conjunctival melanoma and primary acquired melanosis complex was first described in 2007 [15]. So far, there is also no established scheme about the application and duration. Applications of IFN-α2b eye drops over a period of at least 3 months as well as short-term treatments over 6 weeks, partly also with multiple cycles, were described. Over the last 15 years, evidence remained limited in treating conjunctival melanocytic malignancies.

Herein, we present all our cases of histopathological proven CM and PAM, which were treated topically with IFN-α2b or MMC eye drops. To the best of our knowledge, this is the first study comparing the actual clinical use and efficacy of a treatment with topical IFN-α2b and MMC on patients with conjunctival melanocytic malignancies.

## Patients and methods

This retrospective study was approved by the ethics committee of the Albert-Ludwig University Freiburg, Germany (74/20).

To investigate the real-world efficacy, we reviewed all patients with histopathological diagnosis of CM and PAM that had been treated with topical IFN-α2b and/or MMC at the Eye Center Freiburg from July 1997 until April 2019. We reviewed the medical records until January 2022 and extracted duration of treatment, therapy response, recurrences, time to recurrence, outcome at last follow-up, and side effects.

During the analyzed period, treatment regimens varied based on clinical experience and the standard of care at that particular time. Patients received topical IFN-α2b or MMC either as initial therapy or as change of treatment. The study included nine patients who had been treated with topical recombinant IFN-α2b diluted to 1 million international units per ml (Intron A, Merck Sharp & Dohme, Kenilworth, USA) between February 2008 and September 2017. Patients applied it four times daily for 6 weeks to 3 months (= one “therapy unit”). Twenty-one patients had received MMC 0.02% or 0.04% four times daily (= one “therapy unit”) between July 1997 and April 2019. Until July 2002, MMC had been prescribed as a continuous application between 2 weeks to 3 months, afterwards as a cyclic treatment with two or three cycles, each consisting of a 2-week application period and a 2-week break. Four patients were additionally treated with radiation, in two cases simultaneously with IFN-α2b.

In the clinical routine, the diagnosis of recurrence was made either clinically and histologically or clinically alone. Time until recurrence was defined as time between initiation of adjuvant therapy and occurrence of recurrence.

**Fig. 1:**
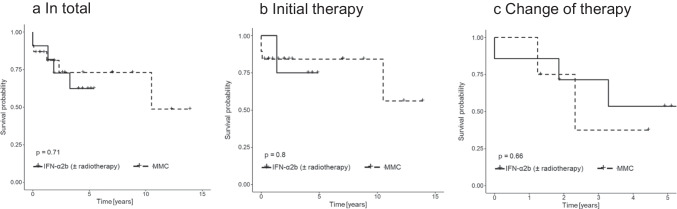
Kaplan–Meier survival curves comparing the efficacy of interferon alpha 2b (IFN-α2b) and mitomycin C (MMC). Survival was defined as absence of recurrence or not complete response. **a** In total (each therapy unit was counted separately). **b** Initial therapy. **c** Change of therapy

Statistical analyses were performed using R Core Team (2020) (R: A language and environment for statistical computing. R Foundation for Statistical Computing, Vienna, Austria. URL https://www.R-project.org/). Efficacy of IFN-α2b and MMC was compared using the log-rank test and visualized using Kaplan–Meier curves (Fig. 1).

## Results

**Fig. 2:**
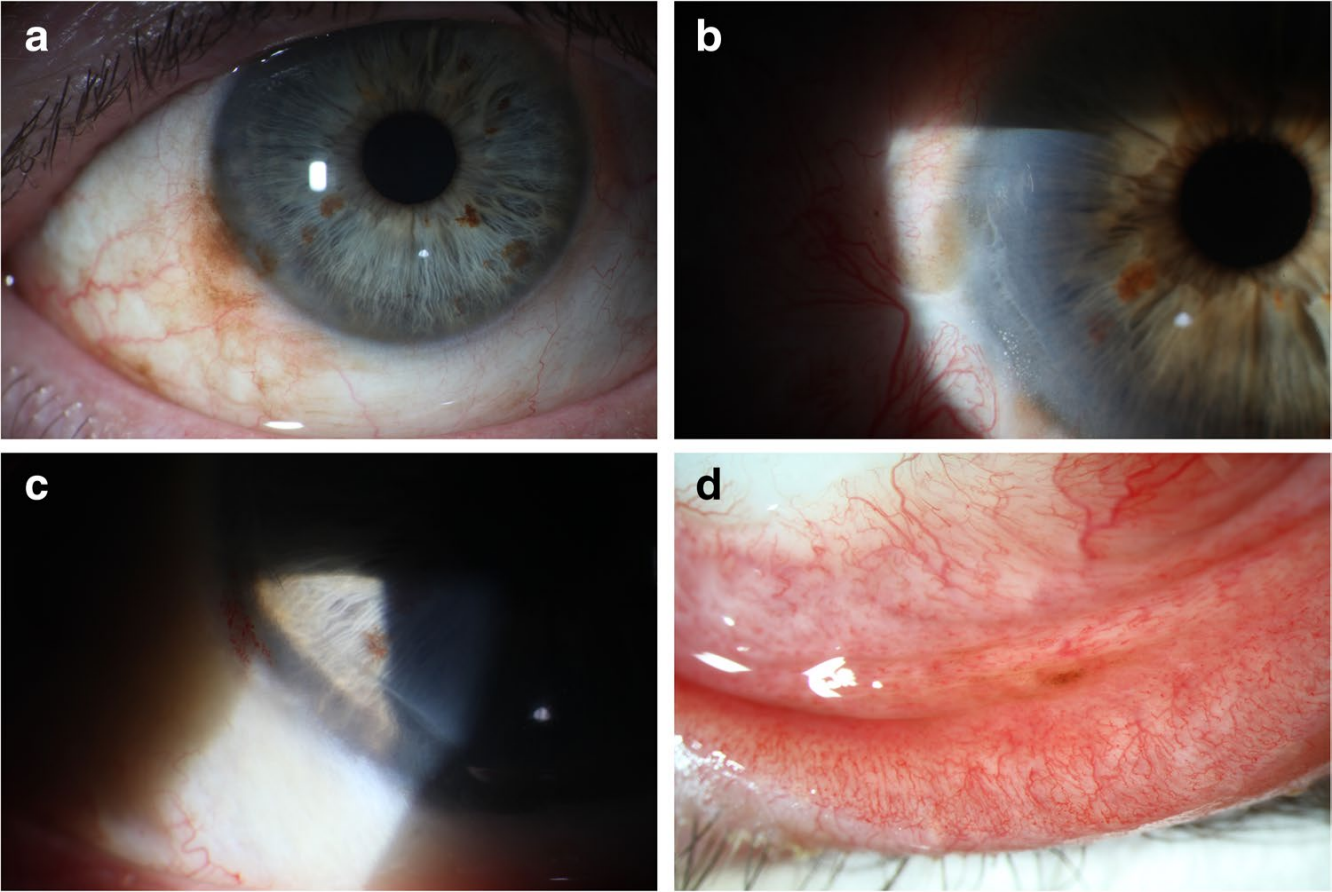
Exemplary clinical presentation of patient 4 (**a**–**d**). **a** Preoperative photograph showing a pigmented conjunctival and corneal lesion. Histological examination after initial excision and mitomycin C (MMC) intraoperatively showed melanoma in situ R1. **b** Early recurrence after radiotherapy. **c** No pigmentation was seen after re-excision with MMC intraoperatively and adjuvant interferon alpha 2b (IFN-α2b) eye drops for 6 weeks. **d** A second recurrence more than 3 years after the first topical IFN-α2b therapy was located in the lower fornix

This retrospective study included 25 patients. Of these patients, two had PAM with atypia, four melanoma in situ, and 19 CM at initial diagnosis. Recurrences were reported in one patient who had PAM with atypia, in one who had melanoma in situ, and in four who had CM at initial diagnosis. Eighteen patients were female and seven male. Age at initial diagnosis ranged between 26 and 85 years (median 66 years). The clinical course of all patients is shown in Table 1. No systemic metastasis was seen in any of these patients. “Not complete response” which was shown in three cases includes cases with incomplete response and non-response. Here, a change of therapy was carried out based on histology twice and once because of persistent pigment clinically. Efficacy of IFN-α2b and MMC is visualized in Fig. 2.

**Table 1:** Clinicopathological features of all 25 patients. *M*, male; *F*, female; *PAM*, primary acquired melanosis with atypia; *M.i.s*, melanoma in situ; *CM*, conjunctival melanoma; *MMC*, mitomycin C; *IFN-α2b*, interferon alpha 2b; * = application three times daily, # = histologically confirmed

Number	Age [years]	Gender	Histopathology (origin)	Adjuvant therapy	Duration	Response	Recurrence [days]	Follow-up [days]	Adverse effect
1	46	M	PAM, R1	MMC 0.02%	2 cycles	Non-response #			
			PAM, R1	IFN-α2b	3 months	Incomplete #			
			PAM, R1	MMC 0.02%	3 cycles	Complete	805		
2	58	F	CM, R1 (PAM)	IFN-α2b	8 weeks	Complete	492		
			M.i.s., R1	MMC 0.02%	3 cycles	Complete		485	
3	47	F	CM, R1 (PAM)	MMC 0.02%	7 cycles	Incomplete			
			-	IFN-α2b	6 weeks	Complete #	679		
			M.i.s, R1	IFN-α2b	3 months	Complete		1798	
4	72	F	M.i.s., R1	Radiotherapy (strontium, 50 Gy)		?	58		
			M.i.s., R1	IFN-α2b	6 weeks	Complete	1201		
			M.i.s., Rx	IFN-α2b	3 months	Complete		1973	
5	77	F	CM, R0 (PAM)	IFN-α2b	6 weeks	Complete		1750	
6	67	F	CM, R1 (PAM)	IFN-α2b + radiotherapy (ruthenium)	6 weeks	Complete		1478	
7	43	F	CM, R0 (PAM)	Radiotherapy (electrons, 50 Gy)	-	Complete	3411		
			CM, R1 (PAM)	MMC 0.02%	3 cycles	Complete	455		
			-	IFN-α2b	3 months	Complete		1839	
8	59	M	CM, R1 (Nevus)	IFN-α2b + radiotherapy (ruthenium)	6 weeks	Complete		1608	
9	78	F	CM, R1 (Nevus)	MMC 0.04%	1 cycle (discontinuation)	Change of therapy			Allergic exanthema
				IFN-α2b	6 weeks	Complete		683	
10	68	F	PAM, R1	MMC 0.02%	3 cycles	Complete		3230	
11	85	F	CM, R1 (Nevus)	MMC 0.02%	3 cycles	Complete		506	
12	75	F	M.i.s., R1	MMC	3 cycles	Complete		2571	
13	71	M	M.i.s., R1	MMC 0.02%	3 cycles	Complete #		947	
14	27	F	CM, R0 (PAM)	MMC 0.02%	3 cycles	Complete		782	
15	41	M	CM, R1 (Nevus)	MMC 0.02%	3 cycles	Complete		99	
16	61	F	CM, R0 (PAM)	MMC	4 weeks	Complete	3844		
			CM, R1 (PAM)	MMC 0.02%	1 cycle (discontinuation)	Complete		1625	Erosion
17	26	F	CM, R1 (Nevus)	MMC 0.02%	3 cycles	Complete #		1038	
18	82	F	CM, R1 (PAM)	MMC 0.02%	3 cycles	Complete		758	
19	66	F	M.i.s., R1	MMC 0.04%	4 cycles	Complete		2505	Limbal stem cell deficiency
20	66	F	CM, R0 (PAM)	MMC 0.04%	3 cycles	Complete		4732	Limbal stem cell deficiency
21	55	F	CM, R0 (Nevus)	MMC 0.02%	1 cycle	Complete		4494	
22	72	F	CM, R1 (PAM)	MMC 0.04%	2 cycles	Complete		233	
23	66	M	CM, R1 (PAM)	MMC*	34 weeks (discontinuation of 1 week)	Complete		239	Pain
24	75	M	CM, Rx (PAM)	MMC 0.02%	3 cycles	Complete		351	
25	84	M	CM, Rx (PAM)	MMC 0.04% *	3 months	Complete		241

### IFN-α2b

In total, nine patients were treated with topical IFN-α2b and two of them twice after recurrences were noted. No side effects occurred in any of these cases. Four patients received IFN-α2b as initial therapy, two of them also with additional radiotherapy. With initial IFN-α2b, all patients showed complete response, three showed no recurrence after a follow-up period of over 3 years (1478, 1608, and 1750 days), and one patient had a recurrence after 492 days. After the therapy was switched to MMC, this patient suffered a further recurrence after 485 days. A change of therapy to IFN-α2b was carried out in five patients, twice because the response to MMC was not complete (non-response and incomplete response), once due to an allergic exanthema after MMC administration, once due to recurrence after radiotherapy (58 days), and once due to a second recurrence after initial radiotherapy (3411 days) followed by MMC (455 days). Of these five patients, one showed incomplete response after showing no response to MMC. Four patients with a change of therapy to IFN-α2b showed complete responses, two with full remission after a follow-up of 683 and 1893 days and two patients suffered from recurrences after 679 and 1201 days. After another administration of IFN-α2b, these two patients showed full remission during a follow-up period of 1798 and 1973 days, respectively.

### MMC

In total, 21 patients were treated with topical MMC and two of them twice after recurrences were noted. MMC 0.02% was prescribed 15 times, 0.04% five times, and in three cases, the concentration was not recorded. Five patients suffered from side effects which included an allergic exanthema (*n* = 1), pain (*n* = 1), erosion (*n* = 1), and limbal insufficiency (*n* = 2) with need of corneal transplantation in one case. The patients with subsequent limbal insufficiency and allergic exanthema have received MMC 0.04%. Nineteen patients received MMC as initial therapy, one showed no response, one showed incomplete response, and in one patient, the therapy had to be discontinued due to an allergic exanthema. In 16 patients with complete responses to MMC as initial therapy, no recurrence was reported in 15 patients with a median follow-up period of 782 days (99–4732 days), five of them with loss to follow-up prior to 1 year. One patient suffered from recurrence after more than 10 years. After another administration of MMC, this patient remained tumor-free (follow-up 1625 days). A change of therapy to MMC was carried out in three patients, once because the responses to MMC and IFN-α2b before were non-response and incomplete and twice due to recurrences after IFN-α2b (492 days) and radiotherapy (3411 days). Of these three patients, all showed complete responses, full remission was reported in one patient (follow-up 485 days) and two patients suffered from recurrences after 805 and 3844 days.

## Discussion

Various adjuvant therapies for conjunctival melanocytic malignancies have been described including topical MMC and more recently IFN-α2b. Due to the documented adverse effects of MMC such as pain and, with more severe consequences, limbal stem cell deficiency, there has been interest in IFN-α2b. To the best of our knowledge, this is the first published study demonstrating and comparing the actual clinical use of topical IFN-α2b and MMC in patients with histopathologically proven CM and PAM.

Evidence for appropriate use and effectiveness of topical IFN-α2b in conjunctival melanocytic malignancies is limited. Furthermore, comparing the documented results is difficult due to a heterogeneous application regime. Altogether, there have been 30 reported cases of CM and 12 of PAM treated with topical IFN-α2b in the literature [15–24]. The largest case series included 12 patients [20]. An adjuvant treatment with topical IFN-α2b was mentioned in 20 cases of CM in a multicenter international data-sharing study, duplications were not ruled out [2]. In the reported 42 cases of CM and PAM treated with topical IFN-α2b, seven cases showed no or incomplete responses. Local recurrences occurred in seven cases and in 27 cases, full remission was mentioned. This goes in line with our results as complete remission was observed in seven of 11 cases treated with IFN-α2b. Unfortunately, there have been mixed results in the previous publications and an aggregation of these studies seems difficult. Moreover, only one case series included patients treated with adjuvant MMC prior to adjuvant IFN-α2b [15]. In three additional case reports, patients were treated with IFN-α2b after topical MMC as primary therapy without prior excision [18, 19, 23]. However, the treatment regimen in our study also varied according to changing clinical experience over time. In addition, both recurrent diseases and primary therapies of recurrences treated with IFN-α2b were included in our study. Therefore, a comparison may still be possible. However, due to changing clinical experience over time, decisions regarding treatment modalities and shifts cannot be generalized and inferred, leading to further heterogeneity between the two groups in our study.

In the literature, therapies were changed to IFN-α2b because of incomplete responses to MMC [19, 23], MMC intolerance with side effects [15, 18, 23], and recurrences after MMC therapy [15, 18], like in our cases. We observed that rates of not complete responses and tumor recurrence were higher after a change of therapy to IFN-α2b compared to IFN-α2b as initial therapy. Only one of four patients with change of therapy to IFN-α2b due to recurrence or not complete response showed full remission.

One reason for the interest in treating conjunctival melanocytic malignancies with a topical alternative to MMC was its adverse effects. These side effects include not only symptoms like tearing, irritation, and pain caused by hyperemia, keratitis, and corneal erosions which can lead to the discontinuation of the therapy but also more serious complications such as limbal stem cell deficiency [11, 12, 25]. In our records, discontinuation of therapy was noted in three out of 21 patients who were treated with MMC. Reasons were corneal erosion, pain, and allergic exanthema. Two other patients developed limbal stem cell deficiency with the need of limbokeratoplasty in one case. Three patients, including both who developed limbal stem cell deficiencies, received MMC 0.04%. Lichtinger et al. evaluated risk factors for limbal stem cell deficiency resulting from topical treatment of MMC for PAM and recommended a reduced concentration of MMC, a reduction in the overall number of treatment courses, or a reduced length of each course [25]. In comparison, local side effects associated with topical IFN-α2b such as follicular conjunctivitis, irritation, chemosis, and superficial punctate keratopathy with corneal edema are rarely reported [15, 26]. In our study, no patient suffered from local or systemic side effects.

When comparing the efficacy of topical IFN-α2b and MMC, our study showed similar results in clinical courses including therapy responses, recurrences, and complete remission rates as shown in Table 2 and Fig. 2. Comparable results in terms of complete remission were also seen in the subgroup analyses when initial use and change of therapy were assessed separately. Considering the subgroup “initial therapy”, recurrences were more frequent with IFN-α2b compared to MMC, but with initial MMC, there were patients who did not even show a complete response in the first place. It should also be noted that two out of four patients with initial IFN-α2b also received combined radiotherapy, and therefore, the effect cannot be attributed to IFN-α2b alone. Considering the subgroup “change of therapy”, recurrence rates were high in both groups. One could assume that the malignant cells may be more aggressive in cases of recurrence or not complete responses, and that a change of therapy may also be less effective. Nevertheless, complete remission was also observed after switching to IFN-α2b (follow-up 683 and 1893 days) as well as MMC (follow-up 485 days). Furthermore, in five patients who received MMC, follow-up time was under 1 year, whereas follow-up times of patients who received IFN-α2b were over 4 years except in one patient with a follow-up time of almost 2 years. Therefore, recurrence rates in patients who received MMC could be underestimated.

**Table 2:** Comparison of the efficacy of interferon alpha 2b (IFN-α2b) and mitomycin C (MMC). Each therapy unit including repeated administration was counted separately. Example: patient 3 initially received MMC, followed by two therapy units of IFN-α2b. All three therapy units were counted in the section “in total”, MMC was counted in the section “initial therapy”, and the first therapy unit of IFN-α2b was counted in the section “change of therapy”

		Response		Full remission	Recurrence
In total	IFN-α2b ° (*n* = 11) (°: combined radiotherapy *n* = 2)	Complete:	**91% (10/11)**	**64% (7/11)**	**27% (3/11)**
		Not Complete:	9% (1/11)		
	MMC (*n* = 23)	Complete:	**87% (20/23)**	**74% (17/23)**	**13% (3/23)**
		Not Complete:	9% (2/23)		
Initial therapy	IFN-α2b ° (*n* = 4) (°: combined radiotherapy *n* = 2)	Complete:	100% (4/4)	75% (3/4)	25% (1/4)
		Not Complete:	0% (0/4)		
	MMC (*n* = 19)	Complete:	84% (16/19)	79% (15/19)	5% (1/19)
		Not Complete:	11% (2/19)		
Change of therapy	IFN-α2b (*n* = 5)	Complete:	80% (4/5)	40% (2/5)	40% (2/5)
		Not Complete:	20% (1/5)		
	MMC (*n* = 3)	Complete:	100% (3/3)	33% (1/3)	66% (2/3)
		Not Complete:	0% (0/3)		

However, in contrast to the reported literature, the results of a recently published multicenter, retrospective data-sharing study question the benefit of adjuvant therapy in patients with conjunctival melanoma. The treatment outcomes in 288 patients with conjunctival melanoma were reviewed [2]. The use of adjuvant therapy including cryotherapy, plaque brachytherapy, or topical chemotherapy did not significantly affect the rate of local recurrence in this analysis. Local recurrence was seen in 8.5% (*n* = 37/199) with adjuvant therapy and in 23.4% (18/77) without. Of all patients with adjuvant therapy, 6.9% (20 patients) received topical IFN-α2b and 37.1% (107 patients) topical MMC. However, a separate analysis of each adjuvant treatment modality in relation to the local recurrence rate was not performed. The authors also mentioned that a bias towards unfavorable tumors receiving adjuvant therapy cannot be excluded.

We acknowledge that our study has a retrospective design with a limited number of patients. However, this is due to the low incidence of conjunctival melanocytic malignancies and the limited evidence with mixed results using topical IFN-α2b. Moreover, only one study included more patients who were treated adjuvantly with topical IFN-α2b [20].

Unfortunately, the production of IFN-α2b was stopped in 2019 and the drug has not been available for order since 2021 in many countries.

## Conclusion

Our observations suggest that adjuvant topical IFN-α2b, possibly in combination with radiotherapy, could have been an alternative with comparable efficacy but more favorable side effect profile to topical MMC in conjunctival melanocytic malignancies. This was also seen in cases with poor efficacy after MMC or radiotherapy; however, a change in therapy to topical IFN-α2b also appeared to be less effective.
